# SSD - a free software for designing multimeric mono-, bi- and trivalent shRNAs

**DOI:** 10.1590/1678-4685-GMB-2019-0300

**Published:** 2020-03-02

**Authors:** Gabriel José de Carli, Abdon Troche Rotela, Greice Lubini, Danyel Fernandes Contiliani, Nidia Benítez Candia, Thiago S. Depintor, Fabiano Carlos Pinto de Abreu, Zilá Luz Paulino Simões, Danilo Fernández Ríos, Tiago Campos Pereira

**Affiliations:** ^1^ Universidade de São Paulo, Faculdade de Medicina de Ribeirão Preto, Departamento de Genética, Ribeirão Preto, SP, Brazil.; ^2^ Universidad Nacional de Asunción, Facultad de Ciencias Exactas y Naturales, San Lorenzo, Paraguay.; ^3^ Universidad Nacional de Asunción, Facultad Politécnica, San Lorenzo, Paraguay.; ^4^ Universidade de São Paulo, Faculdade de Filosofia, Ciências e Letras de Ribeirão Preto, Departamento de Biologia, Ribeirão Preto, SP, Brazil.

**Keywords:** Gene silencing, siRNA, multimeric, shRNA, free software

## Abstract

RNA interference (RNAi) is a powerful gene silencing technology, widely used in analyses of reverse genetics, development of therapeutic strategies and generation of biotechnological products. Here we present a free software tool for the rational design of RNAi effectors, named siRNA and shRNA designer (SSD). SSD incorporates our previously developed software Strand Analysis to construct template DNAs amenable for the large scale production of mono-, bi- and trivalent multimeric shRNAs, via *in vitro* rolling circle transcription. We tested SSD by creating a trivalent multimeric shRNA against the vitellogenin gene of *Apis mellifera*. RT-qPCR analysis revealed that our molecule promoted a decrease in more than 50% of the target mRNA, in a dose-dependent manner, when compared to the control group. Thus, SSD software allows the easy design of multimeric shRNAs, for single or multiple simultaneous knockdowns, which is especially interesting for studies involving large amounts of double-stranded molecules.

RNA interference (RNAi) is a gene silencing technology (Fire *et al.*, 1998) with broad applications, from reverse genetics and functional genomics (Suzuki *et al.*, 2018), to treating diseases (Matthiesen *et al.*, 2019), combating cancer (Ganesh *et al.*, 2018), generating animal models (Guerreo-Rubio *et al.*, 2019) and biotechnological products (Metwali *et al.*, 2015).

The effector molecule is a short RNA duplex, composed of two strands of approximately 21 nucleotides, with two overhanging bases at the 3’ end (Elbashir *et al.*, 2001). However, several types of precursors can trigger gene silencing, such as long double-stranded RNAs (dsRNAs) from 200 to 800 base pairs (bp) (Guo *et al.*, 2018), hairpin RNAs (hpRNAs) (Hussain *et al.*, 2019), small interfering RNAs (siRNAs) (Guo *et al.*, 2018), short hairpin RNAs (shRNAs) (Wang *et al.*, 2018), among others. Notoriously, an ingenious and inexpensive way to produce shRNAs in large quantities was developed, via *in vitro* transcription of a circularized DNA template, generating multimeric shRNAs (mshRNAs) (Seyhan *et al.*, 2006; Abe *et al.*, 2012). In the last decade, such multimeric shRNAs generated by rolling circle transcription have been used by other groups and have been shown to be an interesting option for RNAi (Wang *et al.*, 2015; Shopsowitz *et al.*, 2016; Wu *et al.*, 2016; Kim *et al.*, 2018).[Bibr B7] [Bibr B8]

Here, we present a software tool for a fast and rational design of siRNAs and mshRNAs named “siRNA and multimeric shRNA designer” (SSD) (Figure 1). SSD incorporates the siRNA design tool from our previously developed software Strand Analysis (Pereira *et al.*, 2007), since the design of mshRNAs requires siRNA sequences as input. Once the siRNA duplex is determined, SSD can generate a DNA sequence (the template) whose transcription will result in the mshRNA. There are three options of mshRNAs (Figure 2). The monovalent mshRNA (Figure 2A) displays only one silencing sequence and is a molecule based on the original mshRNA, as designed by Seyhan *et al.* (2006). The bivalent mshRNA contains two silencing sequences (Figure 2B); and the trivalent, three (Figure 2C). Both bi- and trivalent mshRNAs are suitable for silencing single genes by targeting different sites within the mRNA, thus increasing the chance of knockdown. Alternatively, transcripts from different genes can be targeted with the same mshRNA, enabling double or triple knockdown by bi-/trivalent mshRNAs, respectively.

**Figure 1:**
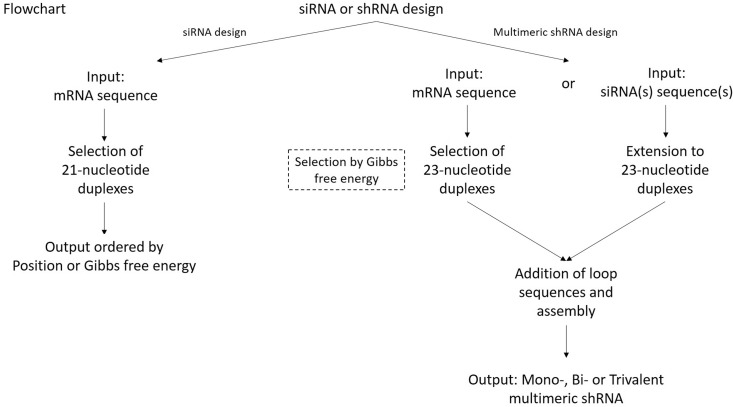
si- and shRNA (SSD) flowchart. SSD software is suitable for designing both siRNAs and multimeric shRNAs. For siRNA design, a target mRNA sequence must be used as ‘input’, while for mshRNA design, either the target mRNA or the previously designed siRNAs sequences can be used as ‘input’. siRNAs may be selected either by ‘position’ within the target mRNA or by `Gibbs free energy’ (i.e., silencing efficiency).

**Figure 2:**
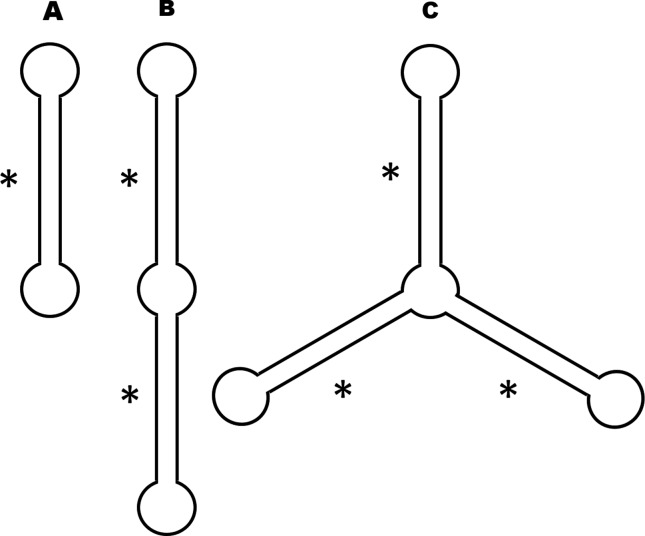
Predicted secondary structures of DNA templates for multimeric shRNAs, designed by SSD against the *Apis mellifera* vitellogenin gene. Circularized templates for mono- (a), bi- (b) and trivalent mshRNAs (c). Each base paired region (*) corresponds to a different shRNA.

In order to make the shRNA design as simple as possible, SSD offers an integrated option to generate the mshRNA template by using the messenger RNA (mRNA) as input. In such case, SSD will select the three most functional and non-overlapping siRNAs to design all the three possible mshRNAs: mono-, bi- or trivalent.

The programming language used to build SSD was Python version 3.5x, because it is a high-level, general-purpose and easily interpreted language. Furthermore, Python has a large, user-friendly standard library, automatic memory management, and dynamic features. The graphical interface tool used was PyQt5, since it provides a high number of prebuilt functions. Moreover, PyQt5 is widely used and has a considerable amount of documentation. One last advantage of Python is that SSD can be executed in most used operating systems (Windows, Mac, and Linux). The URL to download SSD, its requirements and a tutorial detailing how to use the software are on the web pages: https://github.com/bioinf2019/RNA_Tools and https://youtu.be/7pfQ7EVX5w8.

In order to validate SSD functionality, we designed trimeric mshRNAs against the *Apis mellifera* vitellogenin gene (NCBI accession number: NM_001011578.1) and Green Fluorescent Protein (GFP) (NCBI accession number: X83959.1); the latter was used as negative control. The designed linear DNA templates (183-mer) were custom synthesized (Exxtend - Soluções em Oligos, Brazil) and circularized via ligase reactions (20 ng of DNA, 1U of T4 DNA ligase (Promega), in a 10 μL final volume reaction, overnight at room temperature). Subsequently, the entire ligation reaction volume was used for *in vitro* transcription, via TranscriptAid T7 High Yield Transcription Kit (Thermo Scientific), in a final reaction volume of 50 μL, for 4 hours at 37 ºC. In order to remove the excess of free nucleotides after transcription, two precipitation steps with ammonium acetate (5M) were performed. One microliter of mshRNA solution was injected in young adult bees (n = 6-8) between the third and fourth tergites in the abdomen with a microsyringe (Hamilton), in three different concentrations, (i) 250 ng/μL, (ii) 500 ng/μL and (iii) 2500 ng/μL. After injection, bees were maintained at 34 ºC, 80% moisture and unrestricted food (mix of sugar, honey and pollen).

Five days later, total RNA was extracted with Trizol^®^ and 1 μg of RNA was used for reverse transcription reactions (*SuperScript II* ^*®*^, Thermo Fisher Scientific), using oligo(dT)_12-18_ primers (Thermo Fisher Scientific). The cDNA was used in qPCR reactions (qPCRBIO SyGrenn Mix - PCRBIOSYSTEMS) to evaluate the relative expression of the vitellogenin mRNA. The qPCR was performed with the following conditions: 2 min at 95 ºC followed by 40 cycles of 5 s at 95 ºC and 25 s at 60 ºC. The dissociation cycle for every primer pair was from 95 ºC to 60 ºC (15 s each degree), 60 ºC (1 min), and from 60 ºC to 95 ºC (15 s each degree). Vitellogenin specific primers were: GCAGAATACATGGACGGTGT (forward) and GAACAGTCTTCGGAAGCTTG (reverse). As internal control, the gene *RpL32* (Lourenço *et al.*, 2008) was used with specific primers: CGTCATATGTTGCC AACTGGT (forward) and TTGAGCACGTTCAACAAT GG (reverse). qPCR results demonstrate that the trivalent mshRNA designed by SSD was effective in reducing the amount of vitellogenin mRNA in more than 50% (compared to GFP group) when 500 ng of mshRNA were administered (Figure 3). Importantly, 250 ng of mshRNA did not promote efficient knock down, while 2500 ng promoted unspecific effects (data not shown). Such results demonstrate the effectiveness of the current version of SSD, which will be updated once a year, to ensure continuous functionality and improvements.

**Figure 3:**
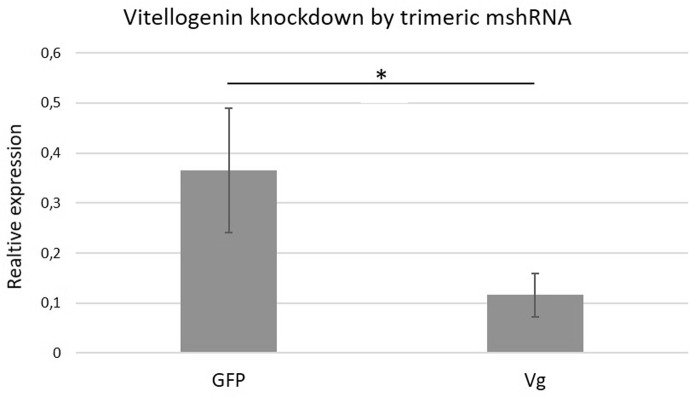
Gene silencing mediated by trivalent multimeric shRNA designed by SSD. GFP group was injected with 500 ng of mshRNAs against GFP gene (negative control); Vg group was injected with 500 ng of mshRNAs against *Apis mellifera* vitellogenin. All experiments were performed in 6-8 biological replicates, each one composed of technical triplicates. Statistical difference was evaluated by ANOVA, with Student T test as post-hoc (* means *p < 0.05*).

Previously, Gvozdeva and colleagues developed a trimeric small-interfering RNA (tsiRNA), which is a linear duplex of RNA with 63 bp, composed of three sequential siRNAs (Gvozdeva *et al.*, 2017). However, due to the impressive yields of *in vitro* transcription systems (up to 7 mg of transcripts per kit), mshRNAs are much cheaper than tsiRNAs, especially in studies using high amounts of RNA duplexes.

Moreover, due to its size (> 30 bp) tsiRNA triggers interferon responses in mammalian cells. Thus, synthesis of tsiRNAs requires modified bases to avoid such unintended secondary effects, which increases the cost of synthesis. In order to be used in mammalian systems, our mshRNAs also demands an additional step - a phosphatase treatment to remove 5’ triphosphate inserted during *in vitro* transcription (Kim *et al.*, 2004). However, such treatment is much cheaper than using modified bases.

Another recent trimeric silencing RNA is the ‘Y-RNA’ (Jang *et al.*, 2018). The structure of Y-RNA is very similar to our trimeric mshRNA (Figure 2C), due to a shared method of synthesis. A circular DNA template undergoes rolling circle transcription by T7 RNA polymerases, generating a long single-stranded RNA with the same secondary structure of our trimeric mshRNA (Figure 2C). However, an additional step is needed - a treatment with RNase H in the presence of an ssDNA helper, in order to promote the cleavage of RNA loops on the edge of the Y-RNA in a site-specific way. According to the authors, the RNase H step facilitates the cleavage of Y-RNA by the nuclease Dicer (Jang *et al.*, 2018). Despite the similarities, the authors do not provide an easy method for a fast and rational design of Y-RNAs; moreover, the RNase H treatment makes the process much more laborious and more expensive when compared to the SSD approach.

In summary, we developed a free software tool (SSD) for rapid design of siRNAs, mono-, bi- and trivalent multimeric shRNAs, which can be easily synthesized in the laboratory, for single or multiple gene knockdowns. An online version of the software will be released shortly.
